# Preface

**DOI:** 10.1093/rpd/ncae205

**Published:** 2024-11-14

**Authors:** Shinji Tokonami, Yoshiya Shimada, Shinji Ueda, James Mc Laughlin, Tibor Kovács

**Affiliations:** Institute of Radiation Emergency Medicine, Hirosaki University, 66-1 Hon-cho, Hirosaki, Aomori 036-8564, Japan; Institute for Environmental Sciences, 1-7 Ienomae, Obuchi, Rokkasho, Aomori 039-3212, Japan; Institute for Environmental Sciences, 1-7 Ienomae, Obuchi, Rokkasho, Aomori 039-3212, Japan; University College Dublin, Belfield, Dublin 4, Ireland; University of Pannonia, Veszprém 8200, Hungary

This special issue of Radiation Protection Dosimetry constitutes the proceedings of International Symposium on Natural and Artificial Radiation Exposures and Radiological Protection Studies (NARE2023), jointly organized by Hirosaki University and Institute for Environmental Sciences. The symposium was held at Hirosaki University 50th Anniversary Auditorium from 19 to 22 September, 2023. The organization of this symposium was sponsored by 14 related companies.

Hirosaki University and the Institute for Environmental Sciences, as core facilities in Japan, are responsible for promoting research and developing human resources in radiological sciences. They have promoted research on natural radiation and biological effects, as well as the environmental and biological impact of the artificial radionuclides resulting from the Fukushima Daiichi Nuclear Power Plant accident. On the other hand, the use of nuclear energy is expected to expand due to the increasing demand for energy and the need to secure stable energy sources. Many nuclear facilities are located around the world, and studies on the impacts of nuclear power under normal operation have been conducted. However, as a result of the global COVID-19 outbreak, which has restricted collaborative research and personnel exchange, the challenge is how to share the latest knowledge on radiation protection with the community for the safe use of radiation.

Therefore, we have decided to organize the “International Symposium on Natural and Artificial Radiation Exposures and Radiological Protection Studies (NARE2023)” face-to-face in Hirosaki, Japan. This symposium covered a variety of radiation research fields focused on radiation exposures and radiological protection studies such as ‘epidemiology’, ‘dosimetry’, ‘high background radiation area’, ‘medical and occupational exposure’, ‘natural and artificial radioactivity’, ‘NORM/TENORM’, ‘radiation biology’, ‘radiation measurement’, ‘radiation protection’, ‘radioactive tracer’ and ‘risk communication’.

A total of 161 scientists from 13 countries in Asia, Africa, and Europe participated in this symposium. The symposium featured 1 keynote lecture, 6 invited talks by internationally renowned researchers, 32 oral presentations, and 114 poster presentations.

This special issue contains 71 peer-reviewed research papers selected from the contributions presented at the symposium. Some work-in-progress presentations were not included but are expected to develop into independent publications in the future.

The organizers sincerely hope that NARE2023 provided a valuable platform for researchers to establish direct connections and foster future collaborations in the fields of natural and artificial radiation exposure, as well as radiological protection studies.

The editors wish to acknowledge the work of the organizers and the committee members for making the NARE2023 symposium possible. A special thank is due to the chairpersons for their cooperation during the symposium sessions and all of the participants for their valuable contributions that made this symposium a success. Furthermore, editing these proceedings were greatly contributed by Naofumi Akata, Kentaro Ariyoshi, Yohei Fujishima, Masahiro Hosoda, Miroslaw Janik, Jun-ichiro Komura, Chutima Kranrod, Tomisato Miura, Eka Djatnika Nugraha, Yasutaka Omori, Minoru Osanai, Yuhi Satoh, Takashi Tani, Hirofumi Tazoe, Shinji Yoshinaga, Hironori Yoshino and Weihai Zhuo. We would also like to express our sincere thanks to all of them.

We gratefully acknowledge the invaluable support and assistance of the local secretariat team throughout all phases of the symposium’s organization. The editors also extend their sincere thanks to the Editorial Board of *Radiation Protection Dosimetry* and the production team at Oxford University Press for their collaboration and efforts in preparing the proceedings.

